# Is HIV index testing and partner notification safe for adolescent girls and young women in low‐ and middle‐income countries?

**DOI:** 10.1002/jia2.25562

**Published:** 2020-08-31

**Authors:** Anne L Stangl, Meroji Sebany, Chisina Kapungu, Cassandra Jessee, Chelsea L Ricker, Eliza Chard

**Affiliations:** ^1^ International Center for Research on Women Washington DC USA; ^2^ Hera Solutions Baltimore MD USA; ^3^ Making Cents International Washington DC USA; ^4^ Independent Consultant Washington DC USA

**Keywords:** adolescent girls and young women, HIV, testing, stigma, violence, gender, LMIC

## Abstract

**Introduction:**

While HIV index testing and partner notification (PN) services have the potential to reach adolescent girls and young women (AGYW) aged 15 to 24 and their sexual partners in need of HIV testing services, the potential social harms have not yet been studied. This commentary highlights the risks of this approach, including intimate partner violence (IPV), stigma and discrimination, and outlines an urgent research agenda to fully understand the potential harms of PN for AGYW, calling for the development of mitigation strategies.

**Discussion:**

A substantial evidence base exists demonstrating the feasibility, acceptability and effectiveness of index testing and partner notification for adults aged 18 years and older in low‐ and middle‐income countries (LMICs), particularly for men, and for adults who are married/cohabiting and referring a current sexual partner. AGYW who are most vulnerable to HIV infection in LMICs do not reflect these demographics. Instead, they are often in age‐disparate partnerships, have limited negotiating power within relationships, experience high rates of violence and face economic challenges that necessitate transactional sex. PN services may be particularly difficult for adolescent girls under 18 who face restrictions on their decision making and are at increased risk of rape. Adolescent girls may also face coercion to notify partners due to unequal power dynamics in the provider–adolescent client relationship, as well as judgemental attitudes towards adolescent sexual activity among providers.

**Conclusions:**

As index testing and PN with AGYW is already being rolled out in some LMICs, research is urgently needed to assess its feasibility and acceptability. Implementation science studies should assess the availability, accessibility, acceptability and quality of HIV PN services for AGYW. Qualitative studies and routine monitoring with age‐disaggregated data are critical to capture potential social harms, PN preferences and support needs for AGYW aged 15 to 17, 18 to 20 and 21 to 24. To mitigate potential harms, PN methods should prioritize confidentiality and avoidance of adverse outcomes. Healthcare providers should be trained to conduct routine enquiry for IPV and provide first‐line support. Support services for AGYW living with HIV and survivors of violence should be implemented alongside HIV PN.

## 1 INTRODUCTION

Adolescent girls and young women (AGYW) are a priority population that may benefit from HIV index testing to ensure early linkage to care and treatment services [1]. Recently, partner notification (PN) linked with HIV index testing has also been recommended for AGYW in low‐ and middle‐ income countries (LMICs) to support global efforts to reach UNAIDS’ 95‐95‐95 goals and achieve epidemic control by 2030 [2]. HIV PN for AGYW is also seen as an entry point for engaging adolescent boys and young men, populations that are harder to reach, in HIV services [3]. However, little is known about the safety of this approach or the potential social harms that AGYW may experience due to HIV PN in LMICs.

Based on the strong evidence of feasibility and acceptability of HIV PN among adults 18 years of age and older, the World Health Organization (WHO) published guidelines for HIV self‐testing and partner notification in 2016 [3]. While HIV PN for adolescents is recommended by WHO, only three studies with adolescents, two qualitative and one observational, were referenced in the guidelines, all of which focused on sexually transmitted infection (STI) PN in high‐income countries [4, 5, 6]. The dearth of evidence continues; there are still no published data on the feasibility and acceptability of this approach in LMICs, especially with adolescent girls aged 15 to 17.

A technical report by YouthPower Learning [7] written by the current authors identified only one additional qualitative study, which examined concerns about and potential barriers to PN among adolescent girls and boys in the United States (U.S.) *if* diagnosed with a STI not including HIV [8]. More recently, a study examined factors associated with successful PN among adolescents aged 13 to 24 living with HIV in 14 U.S. cities. However, the study population was predominantly black men who have sex with men (MSM) whose average age was 21 [9].

Given the paucity of feasibility and acceptability data in LMICs, especially among adolescent girls less than 18, the safety and potential harms of this approach must be considered. This commentary highlights the potential risks, including intimate partner violence (IPV) and stigma, and outlines an urgent research agenda to fully understand the potential harms of PN for AGYW, calling for the development of mitigation strategies.

## 2 DISCUSSION

### 2.1 Types and preferences of partner notification services

HIV PN can be passive, in which clients living with HIV are encouraged to contact their sexual partners directly and inform them that they should be tested, or assisted, where a provider supports the index client to notify their sexual partners [10]. Among adults, assisted referral appears to be the preferred method of notification, and the most effective [7]. A meta‐analysis of three randomized controlled trials (RCTs) conducted by Dalal et al. [10] found that assisted partner notification services led to a 1.5‐fold increase in uptake of HIV testing services (HTS) among partners compared to passive referral. Conversely, studies with adult women and men in Tanzania [11] and youth in the U.S. [9] have reported preferences for passive referral. No studies have examined potential gender or age differences in HIV PN preferences.

Studies on STI PN among youth in high‐income countries have indicated that youth may prefer technology‐facilitated PN, including SMS or text messages, both for convenience and to enhance privacy [4, 6]. However, anonymous provider referral has also been suggested given adolescents’ concerns about their safety and reputation when discussing STI exposures with partners [8]. While we know that different methods of HTS are broadly acceptable to adolescents, including home‐based testing, provider‐initiated testing and, more recently, self‐testing [12], PN is new for this population, so assessing PN preferences, as well as acceptability of PN, is important. In settings where HIV index testing and PN has not yet been rolled out, formative research on PN preferences for AGYW should be conducted to determine the safest methods, which may vary by age and context. Youth engagement is key for implementation science research on adolescent HTS and linkage to care in LMICs [13]. In addition to being study participants, research should actively involve AGYW in the design, implementation and evaluation of the studies to ensure that the insights of adolescents themselves are incorporated into index testing and PN programmes and implementation strategies in order to increase comfort and reduce potential harms.

### 2.2 Is HIV index testing and partner notification safe for AGYW?

In short, we do not yet know if index testing and partner notification will be safe for AGYW in LMICs. While a substantial evidence base exists demonstrating the feasibility, acceptability and effectiveness of index testing and partner notification for adults aged 18 years and older [10], no studies have included girls between the ages of 15 and 17. We also did not find evidence on HIV PN services with transgender women under the age of 18. The average age of participants in eight feasibility studies included in a recent meta‐analysis [10] ranged from 26 [14] to 33 years [11]. Evidence suggests that successful notification and referral are more likely when index clients are male, married or cohabitating, or are referring a current sexual partner [11]. Partner type and quality of the relationship are also predictors of successful PN [9].

However, the AGYW who are most vulnerable to HIV infection in LMICs do not reflect these demographics. Instead, they are often in age‐disparate or short‐term partnerships, have limited negotiating power within relationships, experience high rates of violence and face economic challenges that necessitate transactional sex [15, 16]. PN services may be particularly difficult for adolescent girls under 18, who face restrictions on their decision making and are at increased risk of rape [15, 16] as well as family rejection and social stigma if their HIV status is known. Adolescent girls may also face coercion to notify partners due to unequal power dynamics in the provider–adolescent client relationship, as well as judgemental attitudes towards adolescent sexual activity among providers [17].

As index testing and PN with AGYW is already being rolled out in some LMICs, research is urgently needed to assess its feasibility and acceptability. Implementation science studies with youth engagement [18] should assess the availability, accessibility, acceptability and quality of HIV PN services for AGYW. They should also acknowledge the transience and frequent change in adolescent relationships. Qualitative studies, including participatory methods such as Photovoice [19], and routine monitoring with age‐disaggregated data are critical to capture potential social harms, PN preferences and support needs for AGYW aged 15 to 17, 18 to 20 and 21 to 24. These age cohorts are in different developmental stages, which may translate into different types of partnerships, with different motivations and different social harms related to partner notification.

Structural barriers related to ethical and legal challenges, it should be noted, often stand in the way of rigorous research with adolescents, especially on sensitive topics such as sexual and reproductive health [20]. Given this, the lack of evidence on HIV PN services among AGYW is not surprising. The WHO recently published practical guidance for researchers and reviewers on the most pressing ethical questions [21]. This is an important step in overcoming these barriers and expanding research on sexual and reproductive health with adolescents.

### 2.3 Potential social harms of partner notification

Implementing HIV index testing for AGYW in LMICs could cause undue social harm and impede a healthy transition to adulthood. A range of harms are considered, including anticipated stigma, lack of confidentiality, coercion, risk of IPV and economic ramifications.

#### 2.3.1 Anticipated stigma

Disclosure of HIV serostatus is a key concern of AGYW living with HIV [22, 23]. The anticipation of stigma from peers, family and community members can be severe, and AGYW are often encouraged by parents or guardians not to share their HIV status with anyone outside of their family to protect them from social harms [24, 25]. Access to sexual and reproductive health (SRH) services is often limited, and many AGYW may face additional social and family stigma and consequences if it becomes known that they have begun sexual activity, regardless of whether that sexual activity was consensual [26, 27, 28]. Support services for AGYW living with HIV in LMICs are limited, especially as girls age out of pediatric services and enter adult care and treatment services [29]. Given the dual threat of stigmatization for both sexuality and HIV status, disclosure, even to trusted friends and family, is limited for AGYW living with HIV [30, 31].

Concerns of judgemental attitudes by healthcare providers and a lack of trust in providers have been cited as potential barriers to HIV PN in previous research [32]. Fear of embarrassment, social stigma and shame have also been noted as potential barriers to HIV and STI PN among adults in Barbados and adolescents in the U.S. [8, 32]. In South Africa, one study found that anticipated stigma was the most common stigma‐related barrier to HIV PN [33].

#### 2.3.2 Confidentiality and coercion

The ability to maintain confidentiality in HTS and PN for AGYW is a particular concern. According to UNAIDS, parental consent is required for young people under certain ages before accessing one or more SRH services in 72 countries [34]. Given the varied consent policies in place for adolescents and young people aged 15 to 24 in LMICs, implementing HIV PN with AGYW in this age group may prove challenging [35]. Indeed, recent research suggests that lowering the age of consent for HIV testing may have more of an impact on achieving the UNAIDS 95‐95‐95 targets than any new testing modality [36]. A critical first step in scaling‐up HIV PN for AGYW will be for countries to develop consent policies and practices to facilitate access to and uptake of HTS for adolescents.

Partner notification requires an individual to report details of a partner name and contact information, so it will only be possible if AGYW know the name and contact details of their sexual partner/s. However, unequal power dynamics between healthcare providers and clients, particularly AGYW, and judgemental attitudes towards adolescent sexual activity among providers, may lead to partner notification that is unwanted or unsafe [37]. Women living with HIV are at particular risk of stigma or coercion in the health setting [38, 39], and this same potential exists for AGYW. To limit such harms, providers must receive training on how to offer stigma‐free, gender‐sensitive, youth‐friendly services in a non‐judgemental and supportive manner [40, 41, 42, 43] that reduces coercion.

#### 2.3.3 Risk of intimate partner violence

AGYW are particularly vulnerable to HIV during their early sexual life, in part due to high prevalence of gender‐based violence (GBV) and IPV. A recent study in Kenya found that four in 10 AGYW experienced coerced first sex, and one in nine reported forced first sex [44]. GBV‐associated HIV transmission may be compounded over the sexual life course of AGYW [45] as repeated acts of violence perpetrated by more than one partner are commonly reported [46, 47]. While cases of IPV linked directly to HIV PN have not been reported following HIV PN among adult women in the literature [48, 49, 50, 51], the risk of IPV following HIV PN may be greater for AGYW and must be monitored.

To minimize the potential for IPV among AGYW following PN, healthcare providers must be trained on how to conduct routine enquiry for IPV. Providers should also be trained on the provision of first‐line support for AGYW who disclose experiences or fear of violence. This training should include information on how to utilize new PN tools for screening AGYW for risk of GBV, IPV or other social harms that may result from HIV PN [52]. A list of resources and support services for AGYW living with HIV and their sexual partner/s, as well as survivors of violence, should also be available to providers to facilitate referral.

#### 2.3.4 Economic ramifications

Partnership dissolution, particularly for women fearing loss of economic support, was identified as a key barrier to HIV PN among women in LMICs [32, 53, 54, 55]. Among adolescents, loss of a relationship was also identified as a concern and cited as a key deterrent to notifying a partner about exposure to an STI [8]. The economic ramifications of partnership dissolution for AGYW in LMICs, especially adolescent girls aged 15 to 17, may be especially severe, as they may rely on transactional sex with older men to pay for school fees and daily necessities, such as food and clothing. Given this, AGYW may be hesitant to accept PN. Successfully adapting HIV PN services for AGYW in LMICs may require linkages to structural interventions and social programmes that support girls’ continuation in school (e.g. subsidies for uniforms, cash transfer programmes, etc.) and provide nutritional and housing support to remove the need for transactional sex [16, 56].

### 2.4 Considerations for adapting PN for AGYW

Paramount among considerations regarding the roll‐out of HIV PN for AGYW is ensuring “voluntarism, with informed consent and the explicit right to decline,” as expressly stated in the HIV Self‐Testing and Partner Notification guidance document published by WHO [3]. A range of policy, programmatic and research considerations are recommended prior to or alongside scale‐up of HIV index testing and PN with this vulnerable population (Box 1).

To tailor HIV PN for AGYW, services should ideally be part of a comprehensive package of HIV prevention, care and treatment services that are youth‐friendly [57]. HTS can be an entry point for youth to access other services, such as reproductive health education; peer counselling; life skills development; family planning; diagnosis and treatment of STIs; prevention of vertical transmission of HIV; and mental health and psychosocial support services. Integrating positive youth development (PYD) features into comprehensive HIV services can help support healthy adolescent development, reduce HIV risk behaviours, and address potential barriers and challenges to HIV PN. These features include access to age‐appropriate and youth‐friendly services, life skills‐building, creating safe spaces and building healthy relationships [58].

Box 1 **Key recommendations for ensuring the safety of HIV index testing and partner notification for adolescent girls and young women in low‐ and middle‐income countries.**

Recommendations^a^ for researchers

1. Conduct feasibility and acceptability studies of HIV PN^b^ with AGYW^c^ in LMICs^d^ where implementation is being planned before scaling up HIV PN with this vulnerable population. Where research is underway, encourage age‐disaggregation of data to learn more about young cohorts from ages 15 to 17, 18 to 20 and 21 to 24 years old.
2. Prior to implementing HIV PN for AGYW, countries should conduct rapid situational assessments to understand what PN methods will be safest and most acceptable to AGYW and what resources are available to support AGYW who suffer social harms such as IPV or stigma due to PN.
3. In countries where HIV PN is already being implemented for AGYW, implementation science research to assess the availability, accessibility, acceptability and quality of HIV PN services for AGYW should be conducted and programmes adjusted as needed based on the findings.

Recommendations^a^ for programme implementers

1. Conduct routine programme monitoring of HIV PN services for AGYW once implemented to identify and correct any procedures or processes that may facilitate social harms.
2. Ensure that resources, like gender‐based violence (GBV) support services and social support services for adults and adolescents living with HIV, are in place and prepared to support all adolescents referred from HIV PN services.
3. Alongside the implementation of HIV PN for AGYW, targeted efforts should be made to introduce adolescent boys and young men to HIV testing and increase their engagement in partner referral.
4. Healthcare providers should be trained on:
  - how to conduct routine enquiry for intimate partner violence (IPV), including how to ask about experience or fear of IPV and sexual violence and the provision of first‐line support for AGYW who do disclose experience or fear of violence, including information on how to utilize new tools^e^ to screen AGYW for risk of GBV, IPV or other social harms that may result from HIV PN; and
  - provision of stigma‐free, gender‐sensitive, adolescent‐ and youth‐friendly services in a non‐judgemental and supportive manner.
5. Ensure that HIV PN services are embedded into comprehensive HIV services that are youth‐friendly and integrate positive youth development^f^ features.
6. Implement community mobilization strategies to increase awareness of UNAIDS 95‐95‐95 goals and generate enthusiasm for participating in HIV testing services, including HIV PN.
7. Ensure that adolescent girls and boys have access to information through schools and local community centres and are given the opportunity to build skills on reproductive health to enhance both their knowledge and ability to communicate about reproductive health topics with both healthcare providers and partners.

Recommendation^a^ for policy makers

1. Paramount to the successful implementation of HIV PN for AGYW is the need for countries to develop consent policies and practices to facilitate access to and uptake of HIV testing services among adolescents and ensure confidentiality.

^a^These recommendations were adapted and reprinted with permission from: Stangl A, Sebany M, Kapungu C, *et al*. Technical and Programmatic Considerations for Index Testing and Partner Notification for Adolescent Girls and Young Women: Technical Report. Washington, DC: Youth Power Learning, Making Cents International; 2019. ^b^PN: partner notification; ^c^AGYW: adolescent girls and young women; ^d^LMICs: low‐ and middle‐income countries; ^e^Ricker, C, Stangl, A, Sebany, M, *et al*. (2019) Planning and Conducting Index Testing and Partner Notification for AGYW: Implementation and Clinical Guidance for Health Services; ^f^Positive Youth Development (PYD) features include: access to age‐appropriate and youth‐friendly services, life skills‐building, creating safe spaces and building healthy relationships.

For AGYW, age differentials of sexual partners and unequal power dynamics make disclosure of HIV status particularly challenging. Self‐efficacy is an important predictor of patient‐initiated STI PN for adults and adolescents [59, 60, 61, 62]. Peer support groups and safe spaces can help youth share experiences, address stigma and discrimination and build skills to support partner disclosure. Peer counsellors can serve as trusted and credible sources of support [62]. Integrated youth‐friendly services provide the opportunity for youth‐centred prevention, care and treatment for the multitude of issues affecting youth living with HIV [63].

Strategies for involving adolescent boys and young men in HTS, including index testing and PN, will be critical for minimizing harm for AGYW, who risk being blamed for “spreading HIV” if PN is focused solely on them. Such strategies could include targeted efforts to introduce adolescent boys and young men to HIV testing and increase their engagement in partner referral. Sensitizing the public through mass media campaigns and community mobilization strategies is also recommended to increase awareness of 95‐95‐95 targets and generate broad enthusiasm for participating in HTS, including HIV PN.

Other HIV PN options for AGYW, besides provider‐initiated PN, should be considered for casual partners, including anonymous technology‐facilitated PN (e.g. SMS) or provider referral. HIV PN may not be recommended for unmarried AGYW with few or single partners, as there are real risks of loss of confidentiality due to limited sexual networks. In all cases, HIV PN should only be carried out if preferred by the AGYW and should include follow‐up counselling for all parties.

## 3 CONCLUSIONS

While PN services have the potential to reach AGYW and their sexual partners in need of HIV testing services, the implementation of PN needs careful consideration to minimize potential social harms, particularly for girls under age 18 who may be experiencing violence or stigma, fear violence or stigma, or who may have acquired HIV as a result of violence. PN services for AGYW should also be designed to ensure that AGYW who know their status are linked to appropriate HIV services, such as pre‐exposure prophylaxis (PrEP) to prevent HIV infection, antiretroviral therapy (ART) to suppress HIV viral load, and adherence and social support services for adolescents or partners living with HIV. Such strategies should be incorporated into HIV and SRH services and complemented with the scale‐up of outreach, HIV testing services and PN for adolescent boys, as well as young and adult men.

## COMPETING INTEREST

None declared.

## AUTHORS’ CONTRIBUTIONS

AS, CJ and CK conceptualized the manuscript. MS and CR conducted the literature review. AS led the manuscript writing. MS, CK, CJ, CR and EC were involved in drafting the manuscript and provided critical feedback on the full manuscript. All authors read and approved the final manuscript.
